# Ovarian Metastasis in Endometriod Type Endometrial Cancer

**Published:** 2011-12-22

**Authors:** Mitra Gilani Modaress, Fatemeh Cheraghi, Narges Zamani

**Affiliations:** 1 Department of Gynecology Oncology ,Vali-e-Asr Hospital, Tehran University of Medical Sciences, Tehran, Iran; 2 Department of Gynecology Oncology, Emam Khomeini Hospital, Ahvaz University of Medical Sciences, Ahvaz , Iran; 3 Department of Gynecology Oncology, Tehran University of Medical Sciences, Tehran, Iran

**Keywords:** Endometrial, Cancer, Endometriod Type, Prognosis, Therapy

## Abstract

**Background:**

The purpose of this study was to examine the rate and clinico-Pathological characteristics of ovarian metastasis of endometriod type endometrial cancer.

**Materials and Methods:**

A retrospective study of all patients with endometriod type endometrial cancer was carried out during 1990-2009. Chi-square and Fisher’s Exact tests were used to analyze all the variables. P ≤ 0.05 was considered statistically significant. SPSS software (version 18), was used for statistical analysis of the data obtained.

**Results:**

Two hundred and ten patients fulfilled the inclusion criteria. Only 17 (8%) cases were identified to have ovarian metastasis. Fisherُ s Exact test showed that the independent risk factors of ovarian metastasis in endometrial carcinoma included pathologic grading and depth of myometrial invasion (p<0.001 and p<0.02, respectively).

**Conclusion:**

The risk of ovarian metastasis in the patients with well to moderately differentiated endometriod type endometrial cancer and myometrial invasion limited to less than one half of the myometrium is minimal. Therefore, it is possible to preserve ovaries in young women with early stage endometrioid type endometrial carcinoma, but it is better to remove and freeze ovarian tissue to save fertility without the recurrence risk of ovarian cancer.

## Introduction

In 2008,from the total of 40100 new cases of endometrial cancer ,approximately 7470 deaths were estimated (1, 2).When the endometrial cancer is still confined to the uterus if diagnosed, it has a relatively favorable prognosis compared with other gynecologic malignancies (2). Although the majority of women with endometrial cancer are considered postmenopausal, up to 14% of women are premenopausal (2-7), with 4% under the age of 40 years (2, 3).

The incidence of ovarian metastasis in women with clinical stage I endometrial cancer has been reported by most studies to be approximately 5% (8-10). The standard of care for endometrial cancer includes a hysterectomy and bilateral salpingo- oophorectomy. The decision to preserve the ovaries in young women with endometrial cancer must be carefully justified. Omission of the bilateral salpingo-oophorectomy in young women with endometrial cancer, preferably in those with early stage and low grade disease is offered (11, 12). This strategy offers the potential for future oocyte retrieval as a family-building option while also removing the imminent adverse consequences of estrogen-deprivation (i.e., hotflashes, vaginal atrophy, cardiovascular disease and osteoporosis).

The purpose of this study was to examine the rate and clinic- pathological characteristics of ovarian metastasis of endometriod type endometrial cancer.

## Materials and Methods

From the Tehran Gynecology Oncology ward in Vali-E- Asr Hospital cancer registry data base, were retrospective reviewed the medical records and pathologic reports during the period from 1990 to 2009.

Totally 210 patients fulfilled the criteria and thus they were included in the study. Patients with serous papillary or clear cell tumor histology, with the evidence of extra uterine spread other than to the adenxa, were excluded from further evaluation. All of the patients had a total abdominal hysterectomy and bilateral salpingo - oophorectomy.

This study was approved by the Ethics Committee of Tehran University of Medical Sciences . All information gathered from the hospital records were considered confidential. Statistical analysis was performed using the SPSS software (version 18).

The obtained data from patients with endometriod type endometrial cancer with and without ovarian metastasis were compared in Chi-square and Fisher’s Exact tests. Probability values less than 0.05 were considered as statistically significant.

## Results

The mean age at the time of diagnosis was 53 years (range: 28-72).

Seventeen cases (8.1%) were identified to have ovarian metastasis.

The histologic grade was well differentiated (G1) in 84 (40%) patients, moderately differentiated (G2) in 95 (45.2%) patients and poorly differentiated (G3) in 31 (14.8%) patients.

Eighty five (40.5%) patients had invasion of less than one-half of the myometrial thickness and 125(59.5%) had greater than one-half of the myometrial invasion.

The incidence of ovarian metastasis was 0.00%, 12.6% and 16.1% in patients with well, moderately and poorly differentiated; respectively (p<0.001, Table 1).

In myometrial invasion less than 50%, only 1.2% of the patients had ovarian metastasis , while in invasion greater than 50%, ovarian metastasis was reported in 13.3% of the patients (p<0.02, Table 2).

Ovarian metastasis was the same in both the nulliparous and multiparous women. Also it showed no difference in the premenopausal and menopausal women.

**Table 1:** Ovarian metastasis according to grade


Grade		With ovarian metastasis	Without ovarian metastasis	Total

**3**	Count	5	26	31
	% within grade	16.1%	83.9%	100.0%
**2**	Count	12	83	95
	% within grade	12.6%	87.4%	100.0%
**1**	Count	0	84	84
	% within grade	0.0%	100%	100.0%
**Total**	Count	17	193	210
	% within grade	8.1%	91.9%	100.0%


**Table 2:** Ovarian metastasis according to myometrial invasion


		With ovarian metastasis	Without ovarian metastasis	Total

**≥ 1/2 myometrial invasion**	Count	16	109	125
	within myometr%	13.3%	86.7%	100.0%
**<1/2 myometrial invasion**	Count	1	84	85
	% within myometr	1.2%	98.8%	100.0%
**Total**	Count	17	193	210
	% within myometr	8.1%	91.9%	100.0%


## Discussion

For young women diagnosed with endometrial cancer, possible infertility and estrogen deprivation present difficult challenges for both patients and practitioners. Our data suggest that the risk of ovarian metastasis is low in women with well to moderately differentiated endometriod type endometrial cancer with myometrial invasion less than one half of myometrium.

In a Surveillance, Epidemiology and End Results Database (SEER) analysis by Wright et al. (11)on the safety of ovarian preservation in premenopausal women with stage I endometrial cancer showed , of the 3269 evaluable women, 402 (12%) were found to have had ovarian preservation. Also ovarian preservation had no effect on either cancer-specific survival or overall survival. They concluded that ovarian preservation in premenopausal women with early-stage disease may be safe and not associated with an increase in cancer-related mortality (11).

Zhou et al. suggested that ovarian metastasis rate of patients at clinical stages I and IІ is high, most are concealed and hard to be diagnosed by visual check, and the prognosis of patients with ovarian metastasis is not good. Therefore, one must be careful to retain ovaries of the young endometrial carcinoma patients (12).

Chen and Anderson suggested a reappraisal of the rationale of castration in young patients. They retrospectively reviewed 30 patients with endometrial carcinoma under the age of 40. Ovarian malignancy was seen in only two instances of advanced disease. In their viewpoint, the low risk of ovarian metastasis in young women with stage І disease suggests that thorough surgical staging, hysterectomy with ovarian preservation, is the treatment of choice (13).

Chai et al. believe that many pathologic types of young endometrial carcinoma in patients under 45 are endometrioid adenocarcinoma, related with the long term non-allopathic estrogen stimulation, and that most are combined with hyperplasia of endometrium, and the prognosis is good, especially for patients younger than 35 (14).

Therefore, to young patients at the early stage without high risk factors, if they have no ovarian metastatic by biopsy, we can retain their ovary.

## Conclusion

According to findings of this research, ovarian preservation may be offered to the selected young patients who want to retain ovarian function, with a preoperative histological diagnosis well to moderately differentiated endometriod type endometrial cancer, myometrial invasion limited to less than one half of the myometrium, no gross intraoperative extrauterine tumor spread and no gross abnormality in bilateral ovaries.It is also recommended to the patients who have no inherited predisposition to breast or ovarian cancer. This strategy offers the potential for future oocyte retrieval and can leave the door open for pregnancy from a surrogate mother. Also, with the consent agreement of patient, we can remove and freeze ovarian tissue for possible future use without recurrence risk of ovarian cancer.
